# Urinary Arsenic Metabolites of Subjects Exposed to Elevated Arsenic Present in Coal in Shaanxi Province, China

**DOI:** 10.3390/ijerph8061991

**Published:** 2011-06-03

**Authors:** Jianwei Gao, Jiangping Yu, Linsheng Yang

**Affiliations:** 1 Institute of Geographical Sciences and Natural Resources Research, Chinese Academy of Sciences, 11A Datun Road, Beijing 100101, China; E-Mails: gaojw.06b@igsnrr.ac.cn (J.G.); yujp@igsnrr.ac.cn (J.Y.); 2 Graduate School of the Chinese Academy of Sciences, Beijing 10049, China

**Keywords:** arsenic (As), urinary metabolites, methylation capability, skin lesions, coal arsenic poisoning (CAP), China

## Abstract

In contrast to arsenic (As) poisoning caused by naturally occurring inorganic arsenic-contaminated water consumption, coal arsenic poisoning (CAP) induced by elevated arsenic exposure from coal combustion has rarely been reported. In this study, the concentrations and distributions of urinary arsenic metabolites in 57 volunteers (36 subjects with skin lesions and 21 subjects without skin lesions), who had been exposed to elevated levels of arsenic present in coal in Changshapu village in the south of Shaanxi Province (China), were reported. The urinary arsenic species, including inorganic arsenic (iAs) [arsenite (iAs^III^) and arsenate (iAs^V^)], monomethylarsonic acid (MMA^V^) and dimethylarsinic acid (DMA^V^), were determined by high-performance liquid chromatography (HPLC) combined with inductively coupled plasma mass spectroscopy (ICP-MS). The relative distributions of arsenic species, the primary methylation index (PMI = MMA^V^/iAs) and the secondary methylation index (SMI = DMA^V^/MMA^V^) were calculated to assess the metabolism of arsenic. Subjects with skin lesions had a higher concentration of urinary arsenic and a lower arsenic methylation capability than subjects without skin lesions. Women had a significantly higher methylation capability of arsenic than men, as defined by a higher percent DMA^V^ and SMI in urine among women, which was the one possible interpretation of women with a higher concentration of urinary arsenic but lower susceptibility to skin lesions. The findings suggested that not only the dose of arsenic exposure but also the arsenic methylation capability have an impact on the individual susceptibility to skin lesions induced by coal arsenic exposure.

## 1. Introduction

Arsenic (As) is a ubiquitous element in environment. An established association between human chronic arsenic exposure and a variety of health outcomes, including skin diseases, diabetes, peripheral vascular disease and kinds of cancers [1–5], has been known for many years. It was reported that there was a wide variation in susceptibility to arsenic health outcomes, which may be, to some extent, related to variations in arsenic metabolism [2].

The metabolism of arsenic in humans occurs through reduction and oxidative methylation catalyzed by reductases and methyltransferases [6]. First, pentavalent arsenate is reduced to trivalent arsenite in the blood, and then inorganic arsenic (iAs) is transformed into monomethylarsinic acid (MMA^V^) and dimethylarsinic acid (DMA^V^). Although MMA^V^ and DMA^V^ are less toxic than iAs [6,7], the existence of trivalent intermediates, monomethylarsonous acid (MMA^III^) and dimethylarsinous acid (DMA^III^) during the arsenic metabolism process has been confirmed [8]. They are formed by the reduction of MMA^V^ and DMA^V^ and are more toxic than iAs. Petrick and his colleagues have proposed the following order of toxicity of arsenicals in Chang human hepatocytes: MMA^III^ > arsenite > arsenate > MMA^V^ = DMA^V^ [7]. Therefore, it is considered that methylation is the key to understanding the biotransformation of arsenic and may be related to the mechanism of arsenic toxicity.

Urinary arsenic is generally used as the main biomarker of exposure and is regarded as the most reliable indicator of recent exposure to iAs [9]. A positive correlation between total daily intake of arsenic from water and total arsenic concentration in urine has been found (r = 0.5, *p* < 0.001) in a study from Mexico [10]. The relative distributions of urinary iAs, MMA and DMA have been used as measures of human methylation capability [1,9,11,12]. Besides, primary methylation index (PMI, defined as MMA/iAs) and secondary methylation index (SMI, defined as DMA/MMA) has been applied to assess the first and second methylation step, respectively [1,9,11]. Several studies of arsenism induced by arsenic-contaminated water consumption from Bangladesh, Taiwan and Mexico, have shown an increasing prevalence of arsenic-associated toxic effects with increasing percentage of iAs and MMA^V^ and decreasing percentage of DMA^V^ and SMI [1–3,11].

Worldwide, to date coal arsenic poisoning (CAP) only has been found in China. Shaanxi Province is the second highest CAP-prevalent area next to Guizhou province in China. CAP is caused by arsenic exposure from coal confusion and the coal arsenic concentration is found to be 27.95–475.10 mg/kg (mean: 222.40 mg/kg) in the south of Shaanxi Province [13]. It is much higher than the average arsenic content in coal in the World (9.0 mg/kg) or in China (4.5 mg/kg) [14,15]. Coal is burnt indoors, in stoves without a chimney, for cooking and heating; therefore, indoor air and food were found to be polluted by smoke with high levels of arsenic. The arsenic content of indoor air was 6.32 ± 9.95 μg/m^3^ [13], being 2.3 times higher than the maximum levels of arsenic in the air (3 μg/m^3^) [16]. The average concentrations of arsenic in stove-dried corn and stove-dried chili were 0.76 ± 0.72 mg/kg and 0.96 ± 1.41 mg/kg [13], respectively, which are approximately 3 and 19 times higher than the maximum levels of arsenic allowed in foods in China (0.2 and 0.05 mg/kg, respectively) [17]. Moreover, arsenic concentrations in drinking water [13] were less than 10 μg/L the drinking water standard in China [18]. The CAP situation in Shaanxi Province is serious and the rate of prevalence of CAP ranges from 19.75–70.7% in different villages [19]. In contrast to arsenic (As) poisoning caused by naturally occurring inorganic arsenic-contaminated water consumption, CAP induced by elevated arsenic exposure from coal combustion has been rarely reported, especially in the field of evaluating the association between urinary arsenic profiles and the health outcomes of human.

Within the context of the perspectives mentioned above, the objectives of our study were: (1) to investigate the profile of urinary arsenic metabolites in residents exposed to coal arsenic in the south of Shaanxi Province (China); (2) to find out the relationship between urinary arsenic metabolites and skin lesions induced by chronic arsenic exposure; (3) to evaluate the effect of sex and age on urinary arsenic metabolites and arsenic methylation capability.

## 2. Experimental Section

### 2.1. Study Area

**Figure 1.**
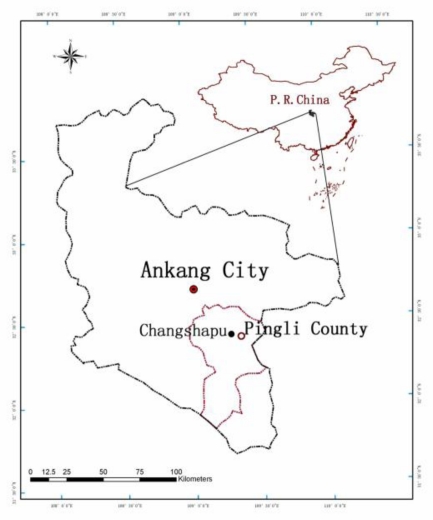
Map of the study area.

The study was conducted in a typical CAP epidemic village, namely Changshapu, in Pingli County of Ankang City located in the south of Shaanxi Province (China) (Figure 1).

Changshapu is a poor small village with an agrarian economy. The climate is usually wet and humid. Therefore, for their daily cooking, heating and indoor drying of grains (corn and chili) the villagers usually burn stone-like coal, which contains a very low content of carbon and a high content of arsenic. The mean concentration of arsenic in the stone-like coal used in Changshapu village was 278 mg/kg [20]. In Changshapu village, the stone-like coal mainly originated from the same colliery near the village, which has been mined for 100 years. Therefore, all the residents had life-long exposures to arsenic polluted air and food.

### 2.2. Study Population

In Changshapu village, there were 335 registered permanent residents, 185 (55%) men and 150 (45%) women. Most of the young people (18 to 40 years old) worked far away from their hometown for most of the year. Therefore, 57 volunteers above eighteen years old participated in this study. They had always lived in Changshapu village since when they were born and had suffered life-long exposure to elevated arsenic concentrations in the environment. In order to identify the cases of skin lesions induced by arsenic exposure, detailed physical examinations were conducted by trained medical doctors according to the Standards of Diagnosis for Endemic Arsenism [21]. In the standards, palms of the hands, soles of the feet and parts of the body trunk were examined for symptoms of skin lesions, including pigmentation, hyperpigmentation (melanosis), hypopigmentation, keratosis, hyperkeratosis, skin ulceration and skin cancers (Bowen disease), and each category of clinical symptoms was assigned a rank. The arsenism patients were then classified as “suspected,” “mild,” “moderate,” “severe,” and “skin cancer” with increasing ranking order. There was only pigmentation and hypopigmentation cases but no keratosis and skin cancers cases among the 57 volunteers. The cases with “suspected”, “mild”, “moderate” and “severe” arsenism were classified as “subjects with skin lesions” and others not showing skin lesions were classified “subjects without skin lesions”. The volunteers included 36 subjects with skin lesions (12 females and 24 males) and 21 subjects without skin lesions (12 females and nine males).

According to the World Medical Association Declaration of Helsinki Ethical Principles for Medical Research Involving Human Subjects [22], all 57 of the volunteers were gave informed consent before participating. The data of personal information, including sex and age, were obtained by questionnaire.

### 2.3. Sample Collection

The information about the heating period in Changshapu village was achieved by inquiry. The residents in Changshapu village started using stone-like coal for heating from November to March each year. Therefore, sample collection was taken in the middle of March, 2008, which was the end of the heating period of Changshapu. According to the IUPAC guidelines of sample collection for trace elements in blood and urine [23], 10 mL of first-morning void urine were obtained in polypropylene tubes and kept in a box with ice. The box was covered to avoid exposure to light. Within 8 h, the urine samples were transported to the local Center of Disease Control and stored at −20 °C. Within five days, all urine samples were transported to the Institute of Geographical Sciences and Natural Resources Research, Chinese Academy of Science for analysis.

### 2.4. Reagents and Standards

The standard solutions of arsenious acid (GBW08666, AsO_3_^3−^), monomethylarsonic acid (GBW08668, CH_3_AsO_3_^2−^) and dimethylarsinic acid (GBW08669, C_2_H_7_AsO_2_) were bought from the National Center of Standard Reference Materials (Beijing, China). The standard solution of arsenate (1,000-mg/L, Na_3_AsO_4_) was from Perkin Elmer Ltd Company (Beijing, China). All other reagents used in this study were guaranteed (the highest grade commercially available in China).

### 2.5. Determination of Arsenic Metabolites

Quantitative determination of urinary arsenic species was performed as described previously [24,25]. The arsenic species (iAs^III^, iAs^V^, MMA^V^ and DMA^V^) in urine were determined by using high-performance liquid chromatography (HPLC) (PerkinElmer 200 series, USA) combined with inductively coupled plasma mass spectroscopy (ICP-MS) (Elan DRC-e, PerkinElmer, USA). The parameters of the instruments for the chromatographic separation and detection were listed in Table 1. Separation was performed on a PRP-X100 anion exchange column (150 mm × 4.10 mm, 5 μm particle size, Hamilton) and a guard column (10 μm particle size, 20 mm length, 2.0 mm i.d., Hamilton). The mobile phase contained 20 mM Ammonium phosphate (dibasic). 15% ammonium hydroxide was used to adjust the pH to 6.0. The mobile phase was filtered through a 0.45 μm membrane and sonicated for 10 min before use in HPLC separation.

**Figure 2.**
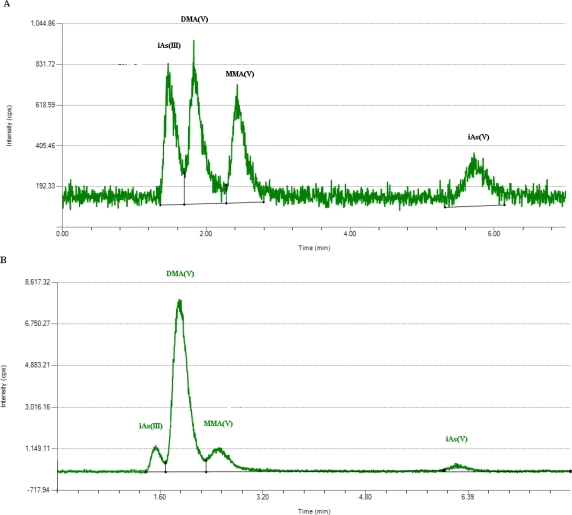
Chromatogram of four arsenic species (**A**) in standard mixture in deionized water; (**B**) in a urine sample.

Briefly, 1-mL urine was passed through a filter with 0.22 μm pore size and 13 mm diameter. Then, each sample was determined by HPLC in triplicate. A typical chromatogram of four arsenic species in standard mixture in deionized water was shown in Figure 2(A) and in a urine sample in Figure 2(B). The retention times of iAs^III^, iAs^V^, MMA^V^ and DMA^V^ were 1.5, 1.8, 2.4 and 5.7 min, respectively.

The Elan DRC-e ICP-MS system was operated in the dynamic reaction cell (DRC) mode. Oxygen was used as the reaction gas. Peak hopping scan mode was used to monitor AsO^+^ at 91 amu. The limits of detection were 0.163 μg/L for iAs^III^, 0.33 μg/L for iAs^V^, 0.15μg/L for MMA^V^ and 0.146 μg/L for DMA^V^. If the concentrations of iAs^V^ in urinary were lower than the limits of detection, the values were recorded as half of the detection limit. The reliability of the arsenic species separation was evaluated by the analytical recoveries of added arsenic species. Spiking the recoveries of iAs^III^, iAs^V^, MMA^V^ and DMA^V^ were 81–95%, 83–98%, 82–102% and 94–110%, respectively.

### 2.6. Creatinine in Urine

Creatinine was necessary for the correction of arsenic concentrations in urinary volume [26]. The concentrations of creatinine were determined by enzyme reaction with a commercial kit (Wako Pure Chemical Industries, Ltd., Japan).

### 2.7. Statistical Analysis

The total arsenic concentration (TAs), the percentages of the four arsenic species, the primary arsenic methylation index (PMI) and the secondary arsenic methylation index (SMI) are calculated as follows:

(1) $$TAs=iAs^{III}+iAs^{V}+MMA^{V}+DMA^{V}$$

(2) $$Percentage\;of\;Arsenic\;species\;(iAs^{III},\;iAs^{V},MMA^{V},DMA^{V})=Arsenic\;species/TAs*100\%;$$

(3) $$PMI=MMA^{V}/(iAs^{III}+iAs^{V})$$

(4) $$SMI=DMA^{V}/MMA^{V}$$

Data analysis was performed by using SPSS software (version 11.5, SPSS Inc., Chicago, IL, USA). The concentrations and percentages of arsenic species, as well as PMI and SMI, were first log-transformed to meet the requirement of equal variance and normal distribution of residuals. The values were transformed back to the arithmetic scale for reporting purposes. The independent samples t-test was used to reflect the difference of the log-transformed data of urinary arsenic species and arsenic methylation capability indexes. Spearman’s correlation was used to analyze the relationships between varies urinary arsenic species and age. The statistical significance was set at *p* < 0.01 and *p* < 0.05, respectively.

## 3. Results and Discussion

### 3.1. Urinary Arsenic Metabolites of the Study Population

The findings regarding the content of arsenic metabolites in urine and the arsenic methylation capability indexes of the 57 volunteers were presented in Table 2. The concentrations of urinary iAs^III^, iAs^V^, MMA^V^, DMA^V^ and TAs were 2.97, 0.9, 4.69, 23.41 and 31.97 μg/g Cr, respectively. DMA^V^ was the major arsenic species in urine (72.38%), followed by MMA^V^ (14.50%), iAs^III^ (9.17%) and iAs^V^ (2.78%). Other two arsenic methylation capability indexes, namely PMI and SMI, were 1.32 and 4.99, respectively.

### 3.2. Urinary Arsenic Metabolites between Subjects with and without Skin Lesions

The concentrations of all of the urinary arsenic species and TAs of subjects with skin lesions were significantly higher than that of subjects without skin lesions (Table 2). It was shown that the subjects with skin lesions excreted more arsenic in urine than subjects without skin lesions, which may indicate a higher recent arsenic exposure in subjects with skin lesions.

The percent DMA^V^ and the SMI were significantly lower, while the percent iAs^V^ was significantly higher of the subjects with skin lesions than that of the subjects without skin lesions. A higher percent iAs^III^ and percent MMA^V^ and a lower PMI were also observed in subjects with skin lesions; however, the difference did not reach statistical significance. These data suggested that the arsenic methylation capability, especially the secondary methylation capability in subjects with skin lesions were lower than in subjects without skin lesions.

### 3.3. Urinary Arsenic Metabolites among Men and Women

The results in Table 3 show that the concentrations of TAs of women were significantly higher than that of men for the subjects without skin lesions. DMA^V^ of women were significantly higher than that of men, whether for the subjects with or without skin lesions. Women may have a higher recent arsenic exposure due to their roles as housewives in families. For the subjects without skin lesions, women had significantly lower percent iAs^III^ and percent MMA^V^, but significantly higher percent DMA^V^ and SMI than men. On the other hand, for the subjects with skin lesions, significantly higher percent DMA^V^ and SMI were observed in women than in men. These differences suggested that arsenic methylation capability, especially the secondary methylation capability of women was higher than that of men.

### 3.4. Relationship between Age and Urinary Arsenic Metabolites

**Figure 3.**
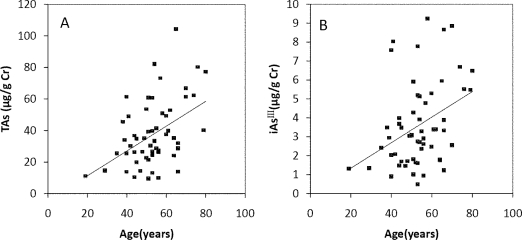

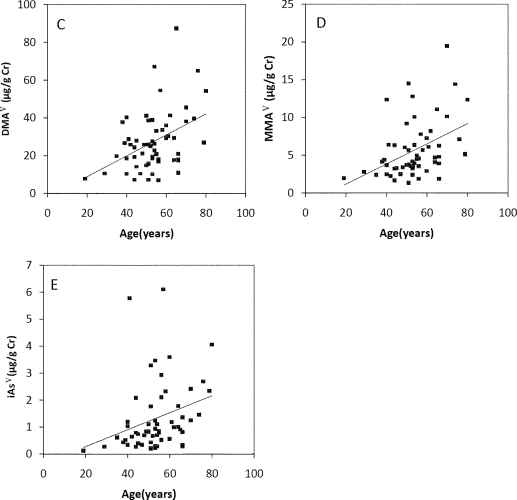
Correlation of age with (**A**) TAs (*R* = 0.411, p = 0.001); (**B**) iAs^III^ (*R* = 0.343, *p* = 0.009); (**C**) iAs^V^ (*R* = 0.422, *p* = 0.001); (**D**) MMA^V^ (*R* = 0.439, *p* = 0.001); (**E**) DMA^V^ (*R* = 0.353, *p* = 0.007) in the urine.

**Figure 4.**
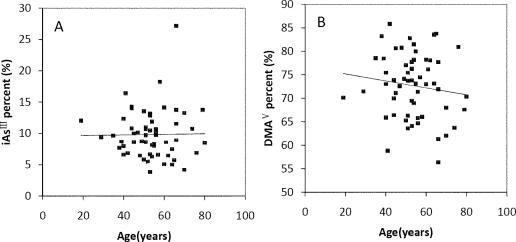

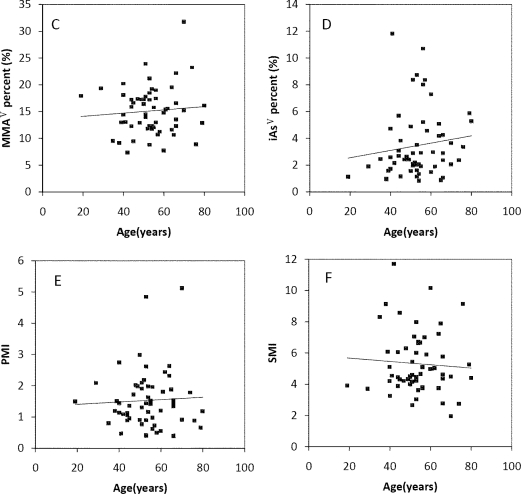
Correlation of age with (**A**) iAs^III^ percent (*R* = −0.06, *p* > 0.05); (**B**) iAs^V^ percent (*R* = 0.195, *p* > 0.05); (**C**) MMA^V^ percent (*R* = −0.17, *p* > 0.05); (**D**) DMA^V^ percent (*R* = −0.133, *p* > 0.05); (**E**) PMI (*R* = −0.021, *p* > 0.05); (**F**) SMI (*R* = −0.007, *p* > 0.05) in the urine.

The Spearman’s correlation coefficients of Age/TAs (0.411), Age/iAs^III^ (0.343), Age/DMA^V^ (0.353), Age/MMA^V^ (0.439) and Age/iAs^V^ (0.422) were shown in Figure 3A–E. The figures shown that age was positive correlated with the concentrations of TAs, iAs^III^, DMA^V^, MMA^V^ and iAs^V^. These suggested that more arsenic may have been accumulated in the elder than in the younger. As regards arsenic methylation capability, the iAs^III^ percent, iAs^V^ percent, MMA^V^ percent, DMA^V^ percent, PMI and SMI did not reach statistical significance with age. (Figure 4). These suggested that the arsenic methylation capability may not correlated with age.

### 3.5. Profiles of Urinary Arsenic Metabolites

Based on population studies, the relative distributions of iAs, MMA^V^ and DMA^V^ in urine of various population groups seems to be fairly constant and in the range of 10–30%, 10–20% and 60–80%, respectively [9,27]. This study clearly showed that the percentages of arsenic species in urine were consistent with previously studies. However, it was documented that there were variations in arsenic methylation between population groups. A lower percent MMA in urine (in the range of 0.0–11%) of Atacamenos living in the Andes [28], whereas an unusual higher percent MMA in urine (20–30% on average) of people living in certain areas of Taiwan were also found [3,29]. The variation of arsenic metabolism between different population groups may be modulated by genetic polymorphism [29–31].

### 3.6. Arsenic Metabolism and Arsenic-Induced Skin Lesions

Urinary arsenic is generally used as the main biomarker of exposure and is regarded as the most reliable indicator of recent exposure to inorganic arsenic [9]. However, it has been reported that dietary intake of seafood, containing high-level organic arsenicals such as arsenobetaine (AsB), arsenocholine (AsC) and arsenosugars, which are regarded as non-toxic, can account for increases of DMA in urine [32]. In this study area, seafood is rare in the diet because of the poor economic conditions and long distance from the sea. Therefore, it was feasible to take total urinary arsenic as a biomarker of exposure to inorganic arsenic. It was shown that the subjects with skin lesions had a higher urinary arsenic excretion (Table 2), indicating a higher recent arsenic exposure than subjects without skin lesions. Dose of arsenic exposure was an important factor being association with its health outcomes. A case-control study from the Black-foot-disease area in Taiwan found that cases with skin cancer had higher total urinary arsenic [33].

The mechanisms of arsenic toxicity are not fully understood. In consideration of the end products in inorganic arsenic methylation being MMA^V^ and DMA^V^, which are less reactive with tissue constituents and are more readily excreted in urine than inorganic arsenic [34], the methylation acts as a detoxification mechanism. However, the trivalent intermediates formed by reduction of MMA^V^ and DMA^V^, namely MMA^III^ and DMA^III^, are more toxic than arsenite [7], and have been found in human urine [35,36]. Because trivalent arsenic species are stronger protein-binders than the pentavalent species [37], MMA^III^ and DMA^III^ generated during the metabolism of arsenic in the cell are the species that interact with cytosolic proteins and may produce some of the toxic effects that accompany exposure to arsenic. Some scholars believe that various urinary arsenic metabolites may be associated with the individual susceptibility to clinical outcomes or diseases induced by arsenic exposure [2,34,37]. A pilot study conducted in Mexico found that the percentages of iAs and MMA^V^ in individuals with cutaneous symptoms were higher than those individuals with normal skin, while the percentage of DMA^V^ was lower [11]. In Bangladesh, a higher proportion of both inorganic arsenic and MMA^V^ and a lower proportion of DMA^V^ in urine were associated with a higher risk of skin lesions after adjusting for age, sex, socioeconomic status, tobacco use and cumulative arsenic exposure [1]. In Taiwan, an elevated urinary MMA^V^ proportion was associated with an increased risk of skin cancer [33]. These literatures from arsenism induced by arsenic polluted water consumption suggested that reduced methylation capability with increased percent MMA^V^, decreased DMA^V^, or decreased DMA^V^/MMA^V^ (SMI) is associated with health outcomes of arsenic exposure. The result of this study conducted in a CAP area of China was consistent with these previous studies. The subjects with skin lesions had a lower arsenic methylation capability, as defined by a higher percent iAs and percent MMA^V^ and a lower percent DMA^V^ and SMI in urine than subjects without skin lesions, which suggested that arsenic methylation capability obviously have an effect on susceptibility to arsenic-induced skin lesions.

It was documented that subjects with a lower percent MMA^V^ in urine may tend to have a lower retention and a faster elimination of ingested arsenic than those with a higher percent MMA^V^ [34]. Higher percent MMA^V^ and lower percent DMA^V^ in urine may be a result of inhibition of the second methylation step, which may increase the chances of cells to be exposed to the more toxic forms of MMA^III^. It is possible that the higher MMA^V^ and lower DMA^V^ in urine are only a marker of higher MMA^III^ in the blood or inside the cells [37]. Besides, MMA^III^ could be oxidized into MMA^V^ rapidly under room temperature conditions [38]. Therefore, it is highly possible that a significant proportion of the measured MMA^V^ is actually derived from MMA^III^. These could be possibly interpretations of people with a lower MMA^V^ and a higher percent DMA^V^ or a higher SMI tending to have a lower risk of developing arsenic induced disease.

### 3.7. Differences of Arsenic Metabolism between Men and Women

In this study, urinary arsenic in men and in women was compared (Table 3). A higher arsenic excretion in urine of women was observed and that may indicate a higher recent arsenic exposure of women. From the results of the face to face questionnaire, it was found that in Changshapu village women usually do housework and spend more time in the kitchen, while men usually cultivate the land and spend more time outdoors. In a study from Shaanxi Province, it was observed that the concentrations of inorganic arsenic in the air of kitchen are higher than in outdoor air [13]. It was possible that women might have more chances to be exposed to arsenic than men do. Therefore, different roles in family taken by men and women might be the reason why the urinary arsenic of women was significantly higher than that of men.

The results had shown that the distributions of arsenic species varied widely among men and women (Table 3). Women had a higher arsenic methylation capability than men in Changshapu village, as defined by significantly higher percent DMA^V^ and SMI in women’s urine. Many other studies have also suggested that the methylation capability of women was better than that of men [1,30,39,40]. This finding was consistent with the results from a study in Guizhou Province, another CAP epidemical area of China [41]. Shraim and his colleagues found that among 51 individuals exposed to arsenic through coal-burning, more than 30% of them had symptoms of arsenism and women had a higher percent DMA but a lower percent iAs in urine. This difference of arsenic methylation capability may be explained by the effect of estrogen. It was believed that estrogens involved in arsenic methylation protect against the skin effects of arsenic [1]. Compared to men, the efficient methylation of arsenic in women might be related to the higher production of choline, which was then oxidized into betaine, the sole alternate methyl group to folate for the remethylation of homocysteine to methionine [42,43]. Elevated homocysteine levels, indicative of a lower one-carbon metabolism, were associated with less efficient methylation of arsenic [40]. Another possible reason for less efficient arsenic methylation in men might be attributed to that men might be exposed to more hazard factors such as smoking [44,45].

According to an epidemiologic investigation conducted in the CAP area in Shaanxi Province, the prevalence of skin lesions in women was lower than men (women *vs.* men: 16.57% and 21.61%, *p* < 0.05) [19]. In this study, it seemed paradoxical but interesting that women had higher urinary arsenic concentrations but lower susceptibility to skin lesions than men. It was shown in Table 3 that similar TAs concentrations were observed in men with skin lesions and women without skin lesions (men with skin lesions: 32.3 μg/g Cr, women without skin lesions: 31.92 μg/g Cr; *p* > 0.1), suggesting that the two groups had well-matched arsenic exposures; but arsenic exposure induced skin lesions in men but did not in women. These data suggested that men showed symptoms of poisoning before women under the same arsenic exposure and that men were more susceptible than women to arsenism. Studies from Bangladesh, Taiwan and India had all reported that men were more susceptible to arsenic-related skin effects than women [46–49]; however, the interpretations were not very clear. Lindberg and his colleagues investigated the interaction between gender and arsenic metabolism for the risk of developing skin lesions and established a hypothesis for the first time that the higher prevalence of arsenic-related skin lesions among men may be mainly attributed to the less efficient of arsenic metabolism among men [1]. In Table 3, women without skin lesions had lower percent MMA^V^ and percent iAs^V^, but higher percent DMA^V^ and SMI than men with skin lesions, indicating that the arsenic methylation capability, particularly the second arsenic methylation capability of women was markedly higher than that of men. These findings approved the hypothesis of Lindberg that women with higher arsenic exposure being less susceptible to skin lesions induced by coal arsenic exposure than men may be explained by the more efficient arsenic methylation of women, as defined by their higher percent DMA^V^and SMI in the urine of women.

### 3.8. Arsenic Excretion and Metabolism in Different Age

In this study, the content of arsenic in urine increased with age, and the arsenic methylation capability was not correlated with age. Age may be associated with arsenic metabolism. A study in Taiwan suggested that a decrease in the second methylation capability may be associated with the increasing of age [50]. A similar diminishing capability with age was also observed by other investigators [3,33]. On the other hand, a case-control study conducted in Taiwan to evaluate the association between exposure to low arsenic levels and urothelial cancer observed no association between age and any of the urinary arsenic indices in patients with urothelial cancer [39]. Aging was negatively associated with SMI in residents of northern Chile, but was not significant when the effect of length of residence in the contaminated area was adjusted [9]. In this study, the residents living in Changshapu had been exposed to arsenic since they were born. The time of duration of arsenic exposure significantly affected the amount of cumulative arsenic exposure. However, the cumulative arsenic exposure did not directly affect arsenic methylation metabolism. Therefore, we could not determine whether there was relationship between age and arsenic methylation interfered by the duration time of arsenic exposure. Furthermore, aging may be associated with a variety of functional changes (such as the liver and kidney) in the organs involved in the metabolism or retention of the metabolites of arsenic [51].

### 3.9. Limitations to the Present Study

Limitations to the present study include the small sample size. Both the use of spot urine samples and the storage of urine samples limited the size of the present sample, and this raises the possibility that findings may have been attributed to chance and hence need to be replicated. However, our sample size is comparable to those of numerous other studies of urinary arsenic metabolism [52,53]. Second, the other important risk factors related with skin lesions such as assets and education were not mentioned in present study, because each person’s assets and education are similar in this poor small village. In future work, we will solve these limitations.

## 4. Conclusions

Subjects with skin lesions had a higher concentration of urinary arsenic and a lower arsenic methylation capability than subjects without skin lesions. Not only the dose of arsenic exposure but also the arsenic methylation capability had an impact on the individual susceptibility to skin lesions induced by coal arsenic exposure. It was found that the capability for inorganic arsenic methylation is modified by sex. The lower susceptibility to skin lesions in women than in men was mainly explained by the more efficient methylation of arsenic, as defined by a higher percent DMA^V^ and SMI in urine among women. Further studies with more larger sample sizes, study protocols and consideration of potential confounders need to be done in the future.

## Figures and Tables

**Table 1.** Operating conditions of the HPLC-ICP-MS for arsenic speciation in urine.

***HPLC parameters***	

Column	Anion Exchange, Hamilton PRP-X100 (15 × 4.1 mm i.d.)
Mobile phase	20 mM Ammonium Phosphate (dibasic); pH 6.0
Column temperature	Ambient
Flow rate	1.2 mL/min
Sample injection volume	20 μL
Runtime	8 min

***ICP Operating parameters***	

Plasma power	1,350 w
Auxiliary gas flow	1.2 L/min
Plasma gas flow	15 L/min
Nebulizer gas flow	1 l L/min
DRC gas flow	0.45 L/min
RPq	0.5

***Mass spectroscopy acquisition***	

Monitored signal	*m/z* 91
Dwell time	250 ms
Scan mode	Peak hopping
Sweeps/reading	1
Readings/replicate	1,200
Replicates	3

**Table 2.** Concentrations and percentages of arsenic species in urine of the volunteers (N = 57).

**Metabolites and Methylation index**	**Total subjects (n = 57) GM (95% CI)**	**Subjects without skin lesions (n = 21) GM (95% CI)**	**Subjects with skin lesion (n = 36) GM (95% CI)**
TAs (μg/g Cr)	31.97 (34.35–47.54)	26.0 (18.68–63.9)	36.09 ^**^ (35.1–49.82)
iAs^III^ (μg/g Cr)	2.97 (3.33–5.05)	2.33 (1.83–2.97)	3.41 ^**^ (2.7–4.3)
MMA^V^(μg/g Cr)	4.69 (3.99–5.51)	3.47 (2.78–4.35)	5.59 ^***^ (4.55–6.85)
DMA^V^ (μg/g Cr)	23.41 (23.49–32.57)	19.67 (15.29–25.29)	25.91 ^*^ (21.29–31.54)
iAs^V^ (μg/g Cr)	0.9 (0.71–1.14)	0.57 (0.40–0.79)	1.18 ^***^ (0.88–1.59)
Percent iAs^III^ (%)	9.17 (8.31–10.12)	8.98 (8–10.08)	9.28 (8.02–10.73)
Percent MMA^V^ (%)	14.50 (13.4–15.67)	13.36 (11.53–15.49)	15.21 (13.87–16.65)
Percent DMA^V^ (%)	72.38 (70.57–74.24)	75.65 (73.49–77.87)	70.54 ^***^ (68.15–73.03)
Percent iAs^V^ (%)	2.78 (2.34–3.31)	2.18 (1.72–2.76)	3.21 ^**^ (2.54–4.06)
PMI	1.32 (1.14–1.53)	1.37 (1.11–1.69)	1.29 (1.05–1.59)
SMI	4.99 (4.54–5.49)	5.66 (4.77–6.72)	4.64 ^**^ (4.15–5.18)

Abbreviations: CI, confidence interval; Cr, creatinine; GM, geometric mean.

*** The difference is significant at *p* < 0.01 compared with subjects without skin lesions;

** The difference is significant at 0.01 < *p* < 0.05 compared with subjects without skin lesions;

* The difference is significant at 0.05 < *p* < 0.1 compared with subjects without skin lesion.

**Table 3.** Concentrations and percentages of arsenic species in urine of the men and women (N = 57).

**Metablates and Methylation index**	**Men (n = 33)**		**Women (n = 24)**	

	**Men without skin lesions (n = 9) GM (95% CI)**	**Men with skin lesions (n = 24) GM (95% CI)**	**Women without skin lesions (n = 12) GM (95% CI)**	**Women with skin lesions (n = 12) GM (95% CI)**

TAs (μg/g Cr)	19.77 (13.27–29.46)	32.3 ^c^ (25.53–40.86)	31.92 ^**^ (23.94–42.58)	47.5 ^a,b^ (33.73–66.89)
iAs^III^ (μg/g Cr)	1.97 (1.32–2.94)	3.24 ^b^ (2.38–4. 4)	2.65 (1.91–3.69)	3.78 (2.56–5.57)
MMA^V^(μg/g Cr)	3.12 (1.98–4.92)	5.16 ^c^ (3.95–6.74)	3.76 (2.89–4.89)	6.55 ^a^ (4.67–9.17)
DMA^V^(μg/g Cr)	14.4 (9.78–21.19)	22.14 ^c^ (17.64–27.8)	24.85 ^**^ (18.31–33.73)	35.49 ^**^ (25.02–50.33)
iAs^V^(μg/g Cr)	0.4 (0.21–0.76)	1.11 ^a^ (0.76–1.6)	0.74 ^*^ (0.52–1.05)	1.34 ^b^ (0.77–2.35)
Percent iAs^III^ (%)	9.96 (9.01–11.02)	10.03 (8.32–12.09)	8.31 ^*^ (6.87–10.05)	7.95 (7.95–6.28)
Percent MMA^V^(%)	15.80 (13.23–18.87)	15.98 ^α^ (14.21–17.96)	11.78 ^**^ (9.53–14.57)	13.78 (11.84–15.97)
Percent DMA^V^(%)	72.81 (69.76–76.00)	68.55 ^c,α^ (66.14–71.06)	77.85 ^**^ (75.19–80.61)	74.70 ^**^ (69.51–80.28)
Percent iAs^V^(%)	2.01 (1.33–3.04)	3.42 ^c,β^ (2.58–4.54)	2.31 (1.66–3.21)	2.83 (1.75–4.56)
PMI	1.48 (1.15–1.90)	1.20 (0.92–1.58)	1.29 (0.91–1.83)	1.49 (1.07–2.07)
SMI	4.61 (3.73–5.69)	4.29 ^α^ (3.76–4.90)	6.61 ^**^ (5.20–8.39)	5.42 ^**^ (4.45–6.61)

Abbreviations: CI, confidence interval; Cr, creatinine; GM, geometric mean.

** The difference is significant at 0.01 < *p* < 0.05 compared with men;

* The difference is significant at 0.05 < *p* < 0.1 compared with men;

a The difference is significant at *p* < 0.01 compared with subjects without skin lesions;

b The difference is significant at 0.01<p<0.05 compared with subjects without skin lesions;

c The difference is significant at 0.05 < *p* < 0.1 compared with subjects without skin lesions;

α The difference is significant at *p* < 0.01 between men with skin lesions and women without skin lesions;

β The difference is significant at 0.05 < *p* < 0.1 between men with skin lesions and women without skin lesions.
